# The Epidemiology of Healthcare-Associated Bloodstream Infection in an Adult Intensive Care Unit: A Retrospective Cohort Study in a Single Tertiary Care Hospital in Hanoi, Vietnam

**DOI:** 10.7759/cureus.31879

**Published:** 2022-11-25

**Authors:** Makoto Inada, Masahiro Ishikane, Nguyen Gia Binh, Mai Lan Huong, Xuan Co Dao, Pham Thi Phuong Thuy, Do Van Thanh, Nozomi Takeshita, Nguyen Quoc Anh, Norio Ohmagari

**Affiliations:** 1 Disease Control and Prevention Center, National Center for Global Health and Medicine, Tokyo, JPN; 2 Intensive Care Unit, Bach Mai Hospital, Hanoi, VNM; 3 Microbiology, Bach Mai Hospital, Hanoi, VNM; 4 National Center for Global Health and Medicine - Bach Mai Hospital (NCGM-BMH) Medical Collaboration Center, Bach Mai Hospital, Hanoi, VNM; 5 Infectious Diseases, Bach Mai Hospital, Hanoi, VNM; 6 Anesthesiology, Bach Mai Hospital, Hanoi, VNM

**Keywords:** healthcare-associated infection, bloodstream infections, low- and middle-income countries, vietnam, hospital epidemiology

## Abstract

Background

Healthcare-associated infections (HAIs), including bloodstream infections (BSIs) in the intensive care unit (ICU), are growing global public health problems. While high-income countries have reported the burden of HAIs precisely, low- and middle-income countries (LMICs), including Vietnam, often lack surveillance systems for HAIs. In Vietnam, few reports described HAI-associated BSIs. Therefore, in this study, we aim to clarify the characteristics of HAI-associated BSI in an adult ICU.

Materials and methods

We conducted a retrospective cohort study of HAI-associated BSI in the adult ICU of Bach Mai Hospital (BMH), Vietnam, between December 2013 and August 2015. For every case identified with bacteremia, we collected characteristics and laboratory findings of the case and followed the length of hospital stay and seven-day and 30-day survival. Predictors of 30-day mortality were analyzed using univariate and multivariate analyses.

Results

Among the 90 cases identified, the median age of the study cohort was 57 (range: 18-90) years, and 59 (65.6%) were male. Chronic heart disease was the most frequent comorbidity (n = 26, 28.9%). The pathogens isolated were mostly *Candida* spp. (n = 26, 26.3%) and *Enterococcus* spp. (n = 19, 19.2%). Among the 90 patients with confirmed HAI-associated BSI, 34 (37.8%) patients survived, while 31 (34.4%) patients died in 30 days. In multivariate analysis, chronic heart disease tended to increase with 30-day all-cause mortality (odds ratio (OR) = 3.5, 95% confidence interval (CI) = 1.0-11.9, p = 0.051).

Conclusions

Our retrospective cohort study is the largest investigation to describe HAI-associated BSI in an adult ICU in a tertiary care hospital in Vietnam. Improved laboratory detection and infection surveillance systems are needed to reduce HAI-associated BSI.

## Introduction

Healthcare-associated infections (HAIs), including bloodstream infections (BSIs) and antimicrobial resistance (AMR), are growing global public health problems [1,2]. HAI could lead to a prolonged hospital stay, increased AMR, high medical expenditure for both the healthcare system and patients, and worse morbidity and mortality. The HAI incidence rate was estimated to be 4.5% per admission, corresponding to 9.3 infections per 1,000 patient days in the USA in 2002 and 71 infections per 1,000 patient days in high-income countries [3,4]. At that time in the USA, urinary tract infections (UTIs) account for 36%, followed by surgical site infections (SSIs) for 20%, and both BSI and pneumonia for 11% [3]. Recent US surveillance in 2016 revealed that 1.8% of the inpatient admission included any diagnosis of HAI [5]. The frequencies of HAI were as follows: *Clostridioides difficile* infection (CDI) accounts for 56%, SSI for 31%, central line-associated BSI (CLABSI) for 7%, catheter-associated UTI (CAUTI) for 4%, and ventilator-associated pneumonia (VAP) for 3% [5].

In general, patients admitted to the intensive care unit (ICU) are vulnerable to HAIs [1]. In high-income countries, approximately 30% of ICU patients are affected by at least one episode of HAIs, with substantial associated morbidity and mortality [6]. VAP is the most frequent cause of infection in the ICU (32%), followed by CAUTI and CLABSI (both 20%) [6]. These HAIs can result in BSI, especially in severe cases. The burden of BSI is high among HAIs, and it is an important cause of mortality, particularly in the ICU.

In high-income countries, there are often national HAI surveillance systems, and data are usually available through national reports and/or scientific literature. On the contrary, in low- and middle-income countries (LMICs), only 15.6% (23/147) of the countries reported functioning national HAI surveillance systems, according to a survey conducted by the World Health Organization (WHO) First Global Patient Safety Challenge in 2005 [7]. Both high-income countries and LMICs suffer from the burden of HAIs, and their rates are higher in LMICs than those in high-income countries [8]. The pooled prevalence of HAI in mixed patient populations in LMICs is reported to be 10.1%, which is higher than the pooled HAI prevalence of 7.6% in high-income countries [1].

Vietnam is an LMIC located in Southeast Asia, with a population of 97.339 million [9], and to the best of our knowledge, there are no studies on national prevalence or yearly incidence of HAI. Single or multicenter studies have been conducted on HAIs or BSIs among ICU patients in Vietnam; however, reports on HAI-associated BSI, especially in the predictors of mortality, are limited [10,11].

Previously, our group showed that the number of blood cultures per 1,000 patient days was lower and the proportion of solitary blood culture sets higher than that of a Japanese study (9.6 versus 25.2 and 49.6% versus 32.8%, respectively), and the proportion of cases of HAI-associated BSI may be relatively high using the retrospective surveillance study in Vietnam [12]. However, in the previous study, the blood culture specimen included both community-acquired and healthcare-related cases, and their clinical prognosis was not analyzed. The purpose of this study was to describe the epidemiology of HAI-associated BSIs in an adult ICU and assess the predictors of 30-day all-cause mortality in a single referential tertiary care hospital in Hanoi, Vietnam.

This article was previously presented as a poster session at the 2017 European Congress of Clinical Microbiology and Infectious Diseases (ECCMID) on April 23, 2017, and as an oral session at the 2017 West Japan Annual Meeting of the Japanese Society of Infectious Diseases on September 26, 2016.

## Materials and methods

Ethics

This study was approved by the Ethics Committee of the National Center for Global Health and Medicine (NCGM) (approval no: NCGM-G-001338-01) and conducted in accordance with the Declaration of Helsinki. Patient data were anonymized before analysis. Informed consent was waived because this study was an observational study and had no intervention. Patients were anonymized before analysis and instead were allotted to a consecutive study ID, which was linked with the hospital ID.

Study design and participants

A single-center retrospective cohort study was performed at Bach Mai Hospital (BMH) (about 1,900 beds), a tertiary and teaching general care hospital in Hanoi, Vietnam, between December 2013 and August 2015. The hospital has services available for consultation with infectious disease (ID) specialists.

Data collection

For each HAI-associated BSI patient, we retrieved the following information: (i) demographic data such as sex and age, (ii) background and comorbid conditions on admission, (iii) recent healthcare-associated exposures, (iv) source of infection, and (v) microbiological data. We also followed all the patients with bacteremia and collected the following information using the link between the hospital ID and the anonymized study ID: (vi) outcomes including seven-day and 30-day all-cause mortality and length of stay (LOS) in the hospital. In addition, we reviewed (vii) referrals to an infectious disease (ID) specialist within 30 days for the management of BSI.

Definition of variables including BSI, HAI, and contamination

An episode of BSI was defined as positive results of microbiological culture of blood samples from a patient with clinical signs and symptoms of infection. Blood culture was considered to be contaminated if the following organisms were identified in only one of the series of blood culture specimens: coagulase-negative *Staphylococci* (CNS), *Propionibacterium acnes*, *Micrococcus* spp., “viridans” group *Streptococci*, *Corynebacterium* spp., and *Bacillus* spp. [12,13]. HAI was defined as any of the following conditions: if the patient (i) was hospitalized in an acute care hospital for more than 48 hours in the previous 90 days, (ii) attended a hospital or hemodialysis clinic, or received intravenous chemotherapy in the previous 30 days, (iii) received intravenous therapy at home or wound care or specialized nursing care in the previous 30 days, and (iv) resided in a nursing home or long-term care facility. BSIs that do not meet the criteria above were considered community-acquired [9,14].

Confirmed CLABSI is defined as BSI occurring 48 hours before or after catheter removal (if any) and positive culture with the same microorganism of either: (i) quantitative central venous catheter (CVC) culture 10^3^ CFU/mL or semiquantitative CVC culture > 15 CFU, (ii) quantitative blood culture ratio CVC blood sample/peripheral blood sample > 5, (iii) differential delay of positivity of blood cultures, which is defined as CVC blood sample culture positive two hours or more before peripheral blood culture (blood samples drawn at the same time), and (iv) positive culture with the same microorganism from pus from the insertion site. Suspected CLABSI is defined as BSI with the presence of a central line and without apparent other sources of infection.

Peripheral line-associated bloodstream infection (PLABSI) is defined as a positive culture with the same microorganism of either: (i) quantitative peripheral venous catheter (PVC) culture 10^3^ CFU/mL or semiquantitative PVC culture > 15 CFU and (ii) positive culture with the same microorganism from pus from the insertion site.

Microbiological data

Bacterial strains from positive blood cultures were identified using the BACTEC™ 9240 Blood Culture System (Becton, Dickinson and Company, Franklin Lakes, NJ, USA) [12]. During the study period, there were no changes in microbiological identification.

Statistical analysis

Continuous variables are shown as median with range or interquartile range (IQR) and were compared using Student’s t-test or Mann-Whitney U test. Categorical variables are shown as absolute and relative frequencies and were compared using the χ^2^ test or Fisher’s exact test. The number of episodes and distribution of BSI sources were described.

Univariate analysis of the predictors of 30-day all-cause mortality was performed using the χ^2^ test or Mann-Whitney U test for each variable. Multivariate analysis of risk factors for 30-day all-cause mortality was constructed using logistic regression. Statistical significance was set as a two-sided p-value < 0.05, and all statistical analyses were performed using Statistical Package for the Social Sciences (SPSS) version 24 (IBM SPSS Statistics, Armonk, NY, USA).

## Results

Participant enrollment process

**Figure 1:**
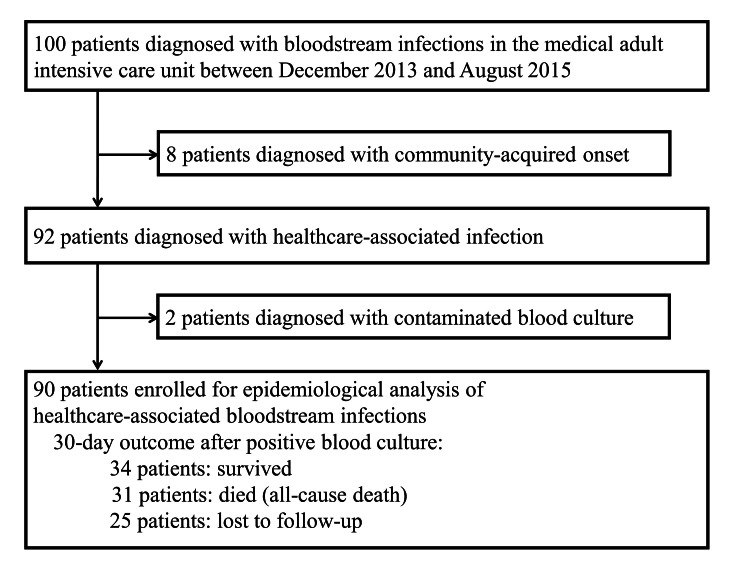
Patient enrollment process

During the study period, 100 patients were diagnosed with BSI in the adult ICU of Bach Mai Hospital between December 2013 and August 2015. We excluded 10 patients diagnosed with community-acquired BSI (n = 8) and those with contamination (n = 2). The remaining 90 patients were enrolled for epidemiological analysis of HAI-associated BSI (Figure 1).

Baseline characteristics of HAI-BSI patients

Among the 90 cases of HAI-associated BSI in the adult ICU, 59 (65.6%) were male, and the median age was 57 (range: 18-90) years. In total, 53 (58.9%) patients had at least one comorbid condition, including chronic heart disease (n = 26, 28.9%), diabetes (n = 22, 24.4%), and chronic kidney disease (n = 16, 17.8%). Overall, 86 (95.6%) patients received healthcare-associated exposure before admission. At the onset of BSI, peripheral line use and central venous line catheter use were observed in 79 (87.8%) cases and 72 (80.0%) cases, respectively, while central line-associated BSI (CLABSI) was confirmed in 22 (24.4%) cases and suspected in five (5.6%) cases. Peripheral line-associated BSI (PLABSI) was diagnosed in two (2.2%) cases (Table 1).

**Table 1:** Baseline characteristics of patients with healthcare-associated bloodstream infections in the adult ICU (n = 90) Unless otherwise stated, data are presented as numbers (%). BSI, bloodstream infection; ICU, intensive care unit; COPD, chronic obstructive pulmonary disease; HIV, human immunodeficiency virus; IV, intravenous; CVC, central venous catheter; CLABSI, central line-associated bloodstream infection; PLABSI, peripheral line-associated bloodstream infection

Variable	Number	(%)
Demographic data		
Sex, male	59	(65.6)
Median age (range)	57	(18-90)
Background and comorbid conditions on admission		
Presence of underlying disease	53	(58.9)
Chronic heart disease	26	(28.9)
Diabetes	22	(24.4)
Chronic kidney disease	16	(17.8)
Steroid use	14	(15.6)
COPD	8	(8.9)
Solid cancer	7	(7.8)
Hematological disease	3	(3.3)
HIV	0	(0)
Biological response modifiers	0	(0)
Healthcare-associated exposure before admission/at the onset of BSI (first positive blood culture)		
Received any healthcare-associated exposure before admission	86	(95.6)
Hospitalized in an acute care hospital (≥2 days) in the previous 90 days	85	(94.4)
Attended a hospital or a hemodialysis clinic, or received intravenous chemotherapy in the previous 30 days	78	(86.7)
Received IV therapy at home or wound care or specialized nursing care in the previous 30 days	5	(5.6)
Resided in a nursing home or long-term care facility	0	(0)
Peripheral line use at BSI onset	79	(87.8)
CVC use at BSI onset	72	(80)
Source of infection		
CLABSI (confirmed)	22	(24.4)
CLABSI (suspected)	5	(5.6)
PLABSI	2	(2.2)
Others	32	(35.6)
Unknown	29	(32.2)

Microbiological and clinical outcome

The median number of days from admission to blood culture was seven (IQR: 2-14) days. A total of 99 microorganisms were isolated from 90 patients. Gram-negative bacteria, gram-positive bacteria, and fungi were identified in 42 (42.4%), 29 (29.3%), and 28 (28.3%) specimens, respectively. The major isolated microorganisms were *Enterococcus faecium* (n = 15, 15.2%), *Escherichia coli* (n = 13, 13.1%), *Candida albicans* (n = 12, 12.1%), *Acinetobacter baumannii* (n = 10, 10.1%), and *Klebsiella pneumoniae* (n = 10, 10.1%) (Table 2).

**Table 2:** Microbiological and clinical outcomes of healthcare-associated bloodstream infections in the ICU (n = 90) Unless otherwise stated, data are presented as numbers (%). BSI, bloodstream infection; ICU, intensive care unit; IQR, interquartile range; MSSA, methicillin-sensitive *Staphylococcus aureus*; MRSA, methicillin-resistant *Staphylococcus aureus*; ID, infectious disease; LOS, length of stay ^1^A total of 99 microorganisms were isolated from 90 patients. ^2^The combination of the polymicrobial infections was as follows: three cases of *E. faecium* and *C. albicans*, two cases of *E. coli* and *C. tropicalis*, a single case of each *E. faecium*and *C. parapsilosis*, *E. faecium* and *A. baumannii*, *E. faecium* and *E. coli*, and *E. faecalis* and *E. coli*.

Variable	Number	(%)
Median days from admission to when blood culture was performed (IQR)	7	(2-14)
Microbiology^1^		
Gram-negative bacteria	42	(42.4)
Escherichia coli	13	(13.1)
Acinetobacter baumannii	10	(10.1)
Klebsiella pneumoniae	10	(10.1)
Burkholderia pseudomallei	2	(2)
Pseudomonas aeruginosa	2	(2)
Serratia marcescens	2	(2)
*Vibrio cholera* non-O1/non-O139	1	(1)
*Ralstonia pickettii*	1	(1)
Group D non-typhoidal *Salmonella*	1	(1)
Gram-positive bacteria	29	(29.3)
Enterococcus faecium	15	(15.2)
Enterococcus faecalis	3	(3)
Enterococcus gallinarum	1	(1)
MSSA	4	(4)
MRSA	3	(3)
* Staphylococcus haemolyticus*	2	(2)
*Corynebacterium* spp.	1	(1)
Fungi	28	(28.3)
Candida albicans	12	(12.1)
Candida tropicalis	9	(9.1)
Candida parapsilosis	2	(2)
Candida guilliermondii	2	(2)
Candida glabrata	1	(1)
Talaromyces (Penicillium) marneffei	1	(1)
* Pichia ohmeri*	1	(1)
Polymicrobial bacteremia^2^	9	(10)
Consulted ID specialist within 30 days of first positive blood culture	48	(53.3)
Outcome		
7-day all-cause mortality after first positive blood culture		
Survived	75	(83.4)
Died	11	(12.2)
Lost to follow-up	4	(4.4)
30-day all-cause mortality after first positive blood culture		
Survived	34	(37.7)
Died	31	(34.4)
Lost to follow-up	25	(27.8)
Median total LOS (days) (IQR)	17	(9-23)
Median LOS after BSI (days) (IQR)	7	(2-14)
Median LOS after BSI excluding those who died (days) (IQR)	9	(4-16)

In total, 48 (53.3%) patients consulted an ID specialist within 30 days of the first positive blood culture. The seven-day and 30-day all-cause mortality rates after the first blood culture were 12.2% (n = 11) and 34.5% (n = 31), respectively. The median total LOS and LOS after BSI were 17 (IQR: 9-23) days and seven (IQR: 2-14) days, respectively (Table 2).

Predictors of 30-day all-cause mortality of HAI-BSI patients

In total, out of 90 patients with HAI-associated BSI, 34 (37.8%) survived and 31 (34.4%) died. Among the 90 patients, predictors of 30-day all-cause mortality of HAI-associated BSI were analyzed for 65 patients, excluding 25 patients who were lost to follow-up.

In the univariate analysis, the presence of any comorbid conditions (odds ratio (OR) = 4.3, 95% confidence interval (CI) = 1.5-12.8, p = 0.006) and chronic heart disease (OR = 3.4, 95% CI = 1.1-10.5, p = 0.032) was associated with 30-day all-cause mortality. However, there were no significant differences in sex, age, source of infection, isolated microorganisms, and consultation with ID specialists between surviving and dead cases. Multivariate analysis showed that a history of chronic heart disease had a tendency to increase with 30-day all-cause mortality (OR = 3.5, 95% CI = 1.0-11.9, p = 0.051) (Table 3).

**Table 3:** Predictors of 30-day all-cause mortality of healthcare-associated bloodstream infections in the ICU (n = 65) Unless otherwise stated, data are presented as numbers (%). BSI, bloodstream infection; ICU, intensive care unit; OR, odds ratio; CI, confidence interval; COPD, chronic obstructive pulmonary disease; CVC, central venous catheter; CLABSI, central line-associated bloodstream infection; IQR, interquartile range; MSSA, methicillin-sensitive *Staphylococcus aureus*; MRSA, methicillin-resistant *Staphylococcus aureus*; ID; infectious disease ^1^CLABSI included definitive (nine died and nine survived) and suspected (three died and two survived). Non-CLABSI included PLABSI (one died and none survived) and others (10 died and 15 survived). Patients with unknown focus were excluded (eight died and eight survived). ^2^A total of 72 microorganisms were isolated from 65 patients.

Variable	Number (%) of patients				Univariate analysis			Multivariate analysis		
	Died		Survived							
	(n = 31)		(n = 34)		Crude OR (95% CI)		p-value	Adjusted OR (95% CI)		p-value
Demographic data										
Sex, male	22	(71)	21	(61.8)	1.5	(0.5-4.3)	0.43	1.9	(0.6-5.8)	0.27
Median age (range)	58	(19-84)	53	(35-63)			0.29	1.0	(1.0-1.0)	0.74
Background and comorbid conditions on admission										
Presence of underlying disease	24	(77.4)	15	(44.1)	4.3	(1.5-12.8)	0.006			
Chronic heart disease	13	(41.9)	6	(17.6)	3.4	(1.1-10.5)	0.032	3.45	(1.00-11.94)	0.05
Diabetes	7	(22.6)	8	(23.5)	1.0	(0.3-3.0)	0.93			
Chronic kidney disease	8	(25.8)	4	(11.8)	2.6	(0.7-3.9)	0.20			
Steroid use	9	(29)	4	(11.8)	3.1	(0.8-11.3)	0.12			
COPD	4	(12.9)	2	(5.9)	2.4	(0.4-14.0)	0.41			
Solid cancer	3	(9.7)	2	(5.9)	1.7	(0.3-11.0)	0.66			
Healthcare-associated exposure before admission/at the onset of BSI (first positive blood culture)										
Received any healthcare-associated exposure before admission	30	(96.8)	33	(97.1)	0.9	(0.1-15.2)	0.95			
Hospitalized in an acute care hospital (≥2 days) in the previous 90 days	30	(96.8)	33	(97.1)	0.9	(0.1-15.2)	0.95			
Attended a hospital or hemodialysis clinic, or received IV chemotherapy in the previous 30 days	28	(90.3)	30	(88.2)	1.2	(0.3-6.1)	0.79			
Received IV therapy at home or wound care or specialized nursing care in the previous 30 days	3	(9.7)	1	(2.9)	3.5	(0.4-35.9)	0.34			
Peripheral line use at BSI onset	28	(90.3)	30	(88.2)	1.2	(0.3-6.1)	0.79			
CVC use at BSI onset	26	(83.9)	29	(85.3)	0.9	(0.2-3.5)	0.87			
Source of infection^1^										
CLABSI versus non-CLABSI	12	(36.4)	11	(32.4)	1.3	(0.4-3.7)	0.59			
Median days from admission to when blood culture was performed (IQR)	9	(3-17)	7	(1-13)			0.12			
Microbiology^2^										
Gram-negative bacteria	14	(45.2)	16	(47.1)	0.9	(0.4-2.5)	0.88			
Escherichia coli	4	(12.9)	6	(17.6)	0.7	(0.2-2.7)	0.74			
Acinetobacter baumannii	4	(12.9)	4	(11.8)	1.1	(0.3-4.9)	1.00			
Klebsiella pneumoniae	3	(9.7)	2	(5.9)	1.7	(0.3-11.0)	0.66			
Burkholderia pseudomallei	0	(0)	1	(2.9)			1.00			
Pseudomonas aeruginosa	2	(6.5)	0	(0)			0.22			
Serratia marcescens	0	(0)	2	(5.9)			0.49			
*Vibrio cholera* non-O1/non-O139	1	(3.2)	0	(0)			0.48			
* Ralstonia pickettii*	0	(0)	1	(2.9)			1,00			
Gram-positive bacteria	9	(29)	9	(26.5)	1.1	(0.4-3.4)	0.82			
* Enterococcus faecium*	5	(16.1)	7	(20.6)	0.7	(0.2-2.6)	0.64			
MSSA	1	(3.2)	1	(2.9)	1.1	(0.1-18.4)	1.00			
MRSA	3	(9.7)	0	(0)			0.10			
*Corynebacterium* spp.	0	(0)	1	(2.9)			1.00			
Fungi	11	(35.5)	13	(38.2)	0.9	(0.3-2.4)	0.82			
Candida albicans	5	(14.7)	5	(16.1)	1.1	(0.3-4.3)	0.87			
Candida tropicalis	4	(12.9)	4	(11.8)	1.1	(0.3-4.9)	1.00			
Candida parapsilosis	0	(0)	2	(5.9)			0.49			
Candida guilliermondii	1	(3.2)	0	(0)			0.48			
Candida glabrata	0	(0)	1	(2.9)			1.00			
Penicillium marneffei	1	(3.2)	0	(0)			0.48			
* Pichia ohmeri*	0	(0)	1	(2.9)			1.00			
Polymicrobial bacteremia	3	(9.7)	4	(11.8)	0.8	(0.2-3.9)	1.00			
Consulted ID specialist within 30 days of first positive blood culture	16	(51.6)	20	(58.8)	0.8	(0.3-2.0)	0.56			

## Discussion

We clarified the epidemiology of microorganisms associated with HAI-associated BSI in the adult ICU of Bach Mai Hospital, Vietnam. The most frequently identified pathogen was *Candida* spp. (26.3%, 26/99), followed by *Enterococcus* spp. (19.2%, 19/99). In a multicenter cross-sectional study conducted in 14 Vietnamese adult ICUs, including Bach Mai Hospital, the most commonly identified pathogens were *Staphylococcus* spp. (27.2%, 12/44), followed by *Acinetobacter baumannii *(22.7%, 10/44) [11]. Another single-center study at the National Hospital for Tropical Diseases, Hanoi, Vietnam, showed that the most common microorganisms in healthcare-associated BSI were *Klebsiella pneumoniae* (28.6%, 22/77) and *Escherichia coli* (11.7%, 9/77) [10]. Several hypotheses can explain these differences. First, because each hospital could have a different environment, different microorganisms might colonize each environment, and patients might be transmitted from the environment. HAI-associated BSIs are thought to be affected by the environment [15]. Second, medical exposure, such as previously prescribed antimicrobials, surgery, and dialysis, to which patients were exposed at each hospital were considered to be different. Although we could not collect this information in our study, these patients had the possibility of being exposed to a lot of medical exposure, such as broad-spectrum antimicrobials, before transfer because Bach Mai Hospital received many severe patients from provincial hospitals in Northern Vietnam. AMR was selected under broad-spectrum antimicrobial pressure, and the fact that the common organisms are* Candida* spp. and *E. faecium* supports this hypothesis [16,17].

We showed that the 30-day all-cause mortality was 34.4% among 90 patients with confirmed HAI-associated BSI. A history of chronic heart disease tended to increase with 30-day all-cause mortality in the multivariate analysis. According to the previous report describing BSI at the National Hospital for Tropical Diseases, Hanoi, Vietnam, all-cause in-hospital case fatality was 110/400 (27.5%), and Cox proportional hazards model analysis revealed that Enterobacteriaceae infections, non-Enterobacteriaceae gram-negative rod (GNR) infections, and the presence of any comorbidities, referred from another hospital, were the factors associated with mortality [10]. Several factors may explain this discrepancy. First, our study analyzed exclusively HAI-associated BSI, while the previous study analyzed both HAI-associated and community-acquired BSI. Second, all of our study population were admitted to the ICU, and the prevalence of comorbidities was 58.9% (53/90), while the population of the previous study includes approximately half of the patients (245/477) admitted to the ICU, and the prevalence of any history of medical condition was 29.8% (142/477). Our study and the previous studies suggest that the prevalence and mortality of HAI-associated BSI in Vietnam or other LMICs might be higher than those in high-income countries [8,18]. Differences in the prevalence and mortality of HAIs between high-income and LMICs were thought to be due to differences in resources such as manpower, HAI surveillance systems, and isolation facilities for appropriate infection prevention and control (IPC) [1,11,19,20]. In LMICs with growing economies, such as Vietnam, rapid population growth requires changes in the healthcare system [20], and there is a need to reduce the prevalence and mortality of HAIs through appropriate IPC.

This study had several limitations. First, it was conducted at the ICU department of a single hospital, Bach Mai Hospital, and it might lack generalizability. This hospital is a tertiary referral hospital located in Northern Vietnam, and many patients are referred from Northern Vietnam. The transferred patients would have been prescribed antimicrobials before the transfer, and the severity of the disease might be high. This might have affected the variation in the identified microorganisms or patient mortality. Our data might be representative of HAI-associated BSI at the ICU in tertiary care hospitals in LMICs rather than general hospitals in LMICs. Second, our data had a relatively small sample size. We included patients exclusively from the ICU department for about two years. This might lead to a lack of power to detect statistical significance. With a more enlarged sample size from other departments or facilities, the data could have more power and generalizability. Third, several data were lacking in this study. The 30-day outcome in 25 patients was unknown due to loss to follow-up. The sources of BSI were classified into CLABSI (either confirmed or suspected), PLABSI, others, or unknown. However, the contents of the others were not well described. We performed univariate and multivariate analyses for 30-day all-cause mortality, but we could not evaluate the time to event due to a lack of data. Lastly, there could be unmeasured confounding factors such as antimicrobial exposure, severity, and treatment. Further studies with detailed evaluations are required.

## Conclusions

Despite the limitations, this is the largest retrospective cohort study of HAI-associated BSI in an adult ICU in a tertiary care hospital in Vietnam. *Candida* spp. and *Enterococcus* spp. are the major pathogens of HAI-associated BSI in the adult ICU. The 30-day all-cause mortality rate was 34.4%, and the history of chronic heart disease was the single risk factor statistically associated with 30-day all-cause mortality in the multivariate analysis. Improved laboratory detection, infection prevention, and control with enhanced and maintained surveillance systems are needed to reduce HAI-associated BSI. Moreover, further studies to compare the changes over time are needed.
